# The role of epigenetic modifications for the pathogenesis of Crohn's disease

**DOI:** 10.1186/s13148-021-01089-3

**Published:** 2021-05-12

**Authors:** M. Hornschuh, E. Wirthgen, M. Wolfien, K. P. Singh, O. Wolkenhauer, J. Däbritz

**Affiliations:** 1 Mucosal Immunology Group, Department of Pediatrics, Rostock University Medical Center, Ernst-Heydemann-Str. 8, 18057 Rostock, Germany; 2 Department of Systems Biology and Bioinformatics, University of Rostock, Universitätsplatz 1, 18055 Rostock, Germany; 3 Stellenbosch Institute of Advanced Study, Stellenbosch University, 7602 Stellenbosch, South Africa; 4 Center for Immunobiology, The Barts and the London School of Medicine and Dentistry, Blizard Institute, Barts Cancer Institute, Queen Mary University, London, UK

**Keywords:** Inflammatory bowel disease, Crohn's disease, Epigenetic modifications, DNA methylation, Epigenetic technologies, Biostatistics

## Abstract

Epigenetics has become a promising field for finding new biomarkers and improving diagnosis, prognosis, and drug response in inflammatory bowel disease. The number of people suffering from inflammatory bowel diseases, especially Crohn's disease, has increased remarkably. Crohn's disease is assumed to be the result of a complex interplay between genetic susceptibility, environmental factors, and altered intestinal microbiota, leading to dysregulation of the innate and adaptive immune response. While many genetic variants have been identified to be associated with Crohn's disease, less is known about the influence of epigenetics in the pathogenesis of this disease. In this review, we provide an overview of current epigenetic studies in Crohn's disease. In particular, we enable a deeper insight into applied bioanalytical and computational tools, as well as a comprehensive update toward the cell-specific evaluation of DNA methylation and histone modifications.

## Background

Crohn's disease (CD) belongs to the group of inflammatory bowel diseases (IBD) and is a relapsing systemic chronic inflammatory disease that affects the gastrointestinal tract with extra-intestinal manifestations and associated immune disorders. Although various therapies have been developed (e.g., immunosuppressants, immunomodulators, biologicals), there is still no cure for CD [1]. It is known that genetic susceptibility is a significant risk factor for the disease. However, environmental factors, such as diet [2], smoking [3], early antibiotic treatment [4], and the intestinal microbiome [5], also play a critical role in the pathogenesis [6]. The importance of lifestyle is supported by the rising incidence of CD in newly industrialized countries in Africa, Asia, and South America [7]. Epigenetic modifications influenced by environmental factors might help to understand the increasing CD incidence [8, 9]. Recently, the analysis of epigenetic changes like DNA methylation or histone modification is recognized as highly useful to identify new biomarkers or targets for drug therapy, which is essential to apply a more targeted treatment in CD [1]. For example, epigenetic alterations in colorectal cancer, allergy and asthma, and cardiovascular disease serve as clinical biomarkers for diagnostic, prognostic, and therapeutic purposes [10–12]. Based on these findings, alterations in DNA methylation between CD patients and healthy controls may likewise be used to identify biomarkers and to improve IBD management.

## Principles of epigenetics

Epigenetics is defined as a change in the organism's phenotype that persists through mitosis and even meiosis without altering the underlying DNA sequence [13]. Consequently, epigenetics is generally understood to be the study of mechanisms that control gene expression in response to environmental influences in a potentially inheritable manner [14]. Complex epigenetic states can be induced by several converging and amplifying signals, including transcription factors, non-coding RNAs, DNA-methylation, and histone modifications. All of these processes are dynamic and reversible. Epigenetic changes are involved in the correct development of cells and their functions, cell differentiation, and homeostasis but are also associated with numerous diseases [13]. Several comprehensive reviews that explained in detail the molecular epigenetic mechanisms have been published on the potential value of epigenetics in IBD during recent years to which we refer the reader [13, 15–17]. In this review, we provide a deeper insight into applied bioanalytical and computational tools for epigenetic analysis, as well as a comprehensive update on CD-specific epigenetic alterations using DNA methylation and histone modifications for biomarker discovery.

### DNA methylation

The most widely studied epigenetic modification in mammals is DNA methylation, which occurs through the covalent bonding of a methyl group to the 5′ carbon of the cytosine residue. When methylation occurs near a promoter sequence, gene expression is inactivated, either because proteins bind to the methylated CpG island and initiate DNA compensation and inactivation or methylation itself blocks the DNA sequence and transcription factors are unable to bind. The conversion of cytosine to 5-methylcytosine is catalyzed by special enzymes called DNA methyltransferases (DNMTs) [18]. In contrast, 5-methylcytosines are converted back to unmethylated cytosines by ten-eleven translocation (TET) proteins with the help of a metabolite intermediate, α-ketoglutarate (α-KG), and molecular oxygen as enzyme cofactors for this reaction [19, 20].

### Histone modification

The DNA themselves is packed together with histones to form chromatin, whereby 147 bp of DNA is wrapped 1.7 times around a core of 8 histone proteins [21, 22]. The N- and C-terminal tails of the nuclear histones stick out from the nucleosome and are responsible for mediating the folding of the chromatin [21]. Various amino acids on the histone tails, namely lysine, arginine, serine, and threonine, are epigenetically altered by enzymes, which then influences if a gene is accessible for binding by transcription factors and the RNA polymerase II machinery. In compact form, named heterochromatin, DNA is less accessible to these factors, so gene expression is suppressed [21]. Conversely, relaxed so-called euchromatin is more accessible, and genes can be transcribed [21, 23]. There are several different classes of histone-modifying enzymes; these modifications include acetylation and deacetylation, methylation and demethylation, phosphorylation, ubiquitination, sumoylation, crotonylation, ADP-ribosylation, and deamination [16, 24].

## Tools for biostatistics

### Epigenetics data analysis and integration of further experimental datasets

The variety of technologies to investigate epigenetic modifications has also led to a broad landscape of data analysis tools, in which the bottleneck has shifted from data generation toward their analysis, posing new challenges for the interpretation of results.

Each of the different platforms to investigate DNA-methylation and histone modification commonly include chip-based arrays, chromatin immunoprecipitation followed by DNA-sequencing (ChIP-Seq), assay for transposase accessible chromatin with high-throughput sequencing (ATAC-Seq), or single-cell technologies that each have its own specific set of analysis tools. In general, all analyses contain data processing steps of quality control, batch correction, bias detection, genetic variation detection (i.e., sequence analysis, peak calling), and differential methylation detection. The Encyclopedia of DNA Elements (ENCODE) consortium performs data curation and also offers standardized processing pipelines for various assay types online (https://www.encodeproject.org/), with regular updates [28]. Specific pitfalls, e.g., during sequence analysis, peak calling, or differential methylation detection, can be obtained in dedicated review articles [29–32]. Novel analysis methods can also be utilized entirely online on dedicated data processing servers, such as Galaxy [33]. A summary of computational protocols used for epigenetic studies in CD is presented in Tables 1 and 2.

**Table 1:** DNA methylation studies on blood samples in Crohn's disease patients

Matrix	Techniques	Results	Limitations	Source
*Whole blood cells*				
WBC	Illumina Human Methylation27 BeadChip system	Methylated loci predominantly involved in immune-related pathways in CD	No differentiation between blood cell subtypes	[54]
PBC	Illumina HumanMethylation450 platform; Illumina TrueSeq Sample Preparation Kit; EpiTexy Bisulfite Kit; EZ-96 DNA Methylation Kit (Zymo)	Samples clustered by disease status and blood cell subtype		[55]
PBC	MassARRAY EpiTYPER platform	*DEFA5* and *TNF* as a speculative biomarker for CD	Only 12 genes investigated	[56]
*Leukocytes and PBMCs*				
PBL	Illumina 450 k DNA methylation analysis	Duplicated results of further GWAS and EWAS in CD for two genes	No differentiation between blood cell subtypes	[57]
PBL	EZ DNA Methylation™ Kit (Zymo); Illumina HumanMethylation450k Bead-Chip Array	Duplicated results of further GWAS and EWAS in CD for 33 genes	Limited sample size, minor effect sizes; systematic meta-analysis necessary	[58]
PBL	Methylation-Specific Amplification Microarray (MSAM); Illumina Infinium HumanMethylation450 BeadChip Kit; EZ DNA Methylation-Gold Kit (Zymo)	No significant differences in DNA methylation between CD and control	Limited sample size; limited coverage/resolution of microarrays employed; clinical heterogeneity of the control and IBD group; no specific cell type investigated	[59]
PBMCs	EZ 96 DNA Methylation Kit; Illumina HumanMethylation450 BeadChip Array	No significant differences in DNA methylation between CD and control	Limited sample size, no comparison between different cell types	[60]
PBMCs	EZ DNA Methylation-Gold kit (Zymo); MethylationEPIC BeadChip (Illumina)	No significant differences in DNA methylation between CD and control	Differentiation between blood cell subtypes	[61]
*Defined cell types*				
B cells	Methylation-Gold Kit (Zymo); GoldenGate Bead Array (Illumina); Bisulfite PCR-based RFLP analysis	Different DNA methylation of immune regulatory genes in CD patients	SNPs or repetitive elements in the probes influenced methylation analysis	[63]
CD8^+^ T cells	Illumina EPIC array	DNA methylation correlated not with disease outcome in CD patients		[64]
CD14^+^ monocytes	EZ DNA Methylation™ kit (Zymo); Illumina HumanMethylation 450 k BeadChip array	Different methylation profiles in monocytes subclasses of CD patients	Limited sample size	[65]
*Serum*				
Human serum	EZ DNA Methylation Kit (Zymo)	57% of the CD patients displayed DNA methylation in *TCERG1L*	Limited clinical information of CD patients; no correlation to healthy controls; only one gene investigated	[62]

*WBCs* whole blood cells, *PBCs* peripheral blood cells, *PBLs* peripheral blood leukocytes, *PBMCs* peripheral blood mononuclear cells, *CD14* cluster of differentiation 14, *CD8* cluster of differentiation 8, *DEFA5* Defensin alpha 5, *TNF* tumor necrosis factor, *TCERG1L* transcription elongation regulator 1-like protein

**Table 2:** DNA methylation studies on tissue samples in Crohn's disease patients

Tissue	Techniques	Results	Limitations	Source
*Biopsies*				
Ascending colon biopsies	EZ DNA Methylation Kit; Methylation-specific PCR (MSP)	Identification of hypermethylated promoters of TSGs in CD	Lack of control samples; no clinical information of included patients; limited sample size, unbalanced ratio (2:1) between male and female patients	[73]
Descending colon biopsies	Illumina Infinium HumanMethylation450 BeadChip Kit; EZ DNA Methylation-Gold Kit (Zymo)	Epigenetically associated gene expression linked to colonic mucosal immune and defense responses in CD	No sufficient statistical power; limited sample size	[67]
Rectum biopsies	EZ DNA Methylation Gold Kit; HumanMethylation27 BeadChip array	Methylation differences reversible after medicinal treatment in CD patients	A limited number of subjects	[74]
*Separated cells from biopsies*				
Human adipose stem cells	Illumina EPIC/850 k array; Gene expression analysis	Different DNA methylation related to the immune system and cell differentiations processes in active and inactive CD; changes in DNA methylation-related gene expression partially restored in quiescent CD	Observational study; limited sample size	[68]
Human intestinal fibroblasts from colon biopsies	MethylMiner Methylated DNA Enrichment Kit; Illumina's ChIP-Seq DNA Sample Prep Kit	The majority of different DNA methylation was within introns and intergenic regions and not associated with CpG islands in CD patients	Tiny patient numbers; little diversity	[75]
Human intestinal fibroblasts of terminal ileal	Methylation analysis Illumina EPIC BeadChip array	Methylation changes depend on inflammation status in the tissue of CD patients	Limited sample size	[78]
Intestinal epithelial cells	Illumina HumanMethylation450; Illumina EPIC bead chips	Gut-segment specific DNA methylation differences in CD patients	Limited sample size	[76]
Intestinal epithelial cells	Illumina Infinium HumanMethylation450 BeadChip	Methylation changes between fetal and pediatric samples indicate importance in embryonal development; IBD-specific intestinal epithelial DNA methylation signature in CD and UC patients	Limited sample size	[79]

*TSGs* tumor suppressor genes

Similar to transcriptomics, epigenetic profiles are continuous, dynamic, and tissue-specific, so the challenges are also more and more focused on data analysis approaches to facilitate the identification of coordinated epigenetic changes and interpretation of their functional consequences in normal development and disease [34]. Therefore, integration of multiple omics datasets might enable an in-depth understanding of the interplay between various cogs of the transcriptional machinery [35], especially an essential asset to investigate CD. For example, the ENCODE database [36] incorporates numerous CD-related datasets on gene expression, ChIP-Seq (transcription factor binding, histone modifications), genome-wide association studies (GWAS), and expression quantitative trait loci (eQTL) obtained from multiple cell types. Successfully applied epigenetics integration studies that utilized RNA-Seq and ChIP-Seq or single-cell RNA-Seq and single-cell ChIP-Seq have already been reported for other diseases [37–39]. The extended use of single-cell RNA-Seq data continues with novel approaches that apply cell type deconvolution to infer bulk tissue DNA methylomes [40]. Interestingly, the epigenetic clock developed by Horvath [41] utilizes DNA methylation data only to provide an estimate of age; however, the testing data used in generating this clock did not have a large representation of tissue from elderly individuals, and as such, it is unclear if the clock is accurate in older age groups or those with age-related diseases [42]. These novel experimental and computational methods can be essential to investigate currently limited cell type-dependent influences, e.g., monocytes, macrophages, in CD patients.

### Application of machine learning algorithms to decipher important epigenetic modifications used for patient classification

Despite the fact that novel experimental and computational technologies have become available, the application to specific CD examples is limited.

Machine learning (ML) and deep learning (DL) are subsets of currently widely used artificial intelligence technologies, in which computer algorithms are used to autonomously learn from given data and information. Current reviews of Hamamoto et al. [43] and Rauschert et al. [44] also foresee integrated epigenetics analyses of “medical big data” or omics data as essential, and both show the general advantages and pitfalls of ML and DL technologies. In total, sixteen studies were identified that utilize ML and epigenetics data to diagnose or classify diseases [44]. Exemplarily for clinical epigenetics in a routine diagnostic setting, Capper et al. already demonstrated the applicability of an ML-based approach for the classification of central nervous system tumors by using DNA-methylation data [45]. This study suggests that such a predictive DNA methylation-based classification model might substantially influence diagnostic precision compared to current standard methods, resulting in a change of diagnosis in up to 12% of prospective cases. Taking into account the growing number of reported epigenetic alterations in diseases, this offers a chance to increase sensitivity and specificity of future diagnostics and therapies in general [44].

### Network modeling and functional analyses for epigenetics data integration

Network-based modeling approaches already present valuable insights to understand and improve the perception of complex disease states and their initial causes. To date, network analysis provides reliable and cost-effective approaches for early disease detection, prediction of co-occurring diseases and interactions, and drug design [46]. For example, gene promoter annotation combined with network analysis and sequence-resolution of enriched transcriptional motifs in epigenetics data can reveal transcription factor families that act synergistically with epigenetic master regulators [47]. Wilson et al. conclude that there is tremendous therapeutic potential in understanding and targeting epigenetic modification pathways [47]. In particular, investigating the cooperation of chromatin remodelers and the transcriptional machinery is not only crucial for elucidating fundamental mechanisms of chromatin regulation but also necessary for the design of targeted therapeutics.

It was also shown that integrating differentially methylated genes, which have been identified with ML-based approaches, and gene expression data in a network context permits greater resolution for cancer-associated genes and pathways than observed previously [48]. Integrational studies with proteomics and epigenetics data can extend common protein–protein interaction networks, which are exclusively focused on protein–protein associations and resulting in cell activities. In particular, Zaghlool et al. [49] were able to link DNA methylation to disease endpoints via intermediate proteomics phenotypes and identify correlative networks that may eventually be targeted in a personalized approach of chronic low-grade inflammation.

Similar to analyses with transcriptomics data, functional enrichment analyses in epigenetics data can be performed by popular approaches, such as EnrichR [50], gProfiler [51], or by using the gometh function from the R missMethyl package [52], to investigate the biological impact of the discovered differentially expressed genes or methylated sites. Correlation studies with clinical outcome data and patient subgrouping can be performed via clustering approaches, e.g., principal component analysis (PCA), or its co-embedded visualizations of t-distributed stochastic neighbor embedding (t-SNE), or uniform manifold approximation and projection (UMAP) analyses.

**Fig. 1:**
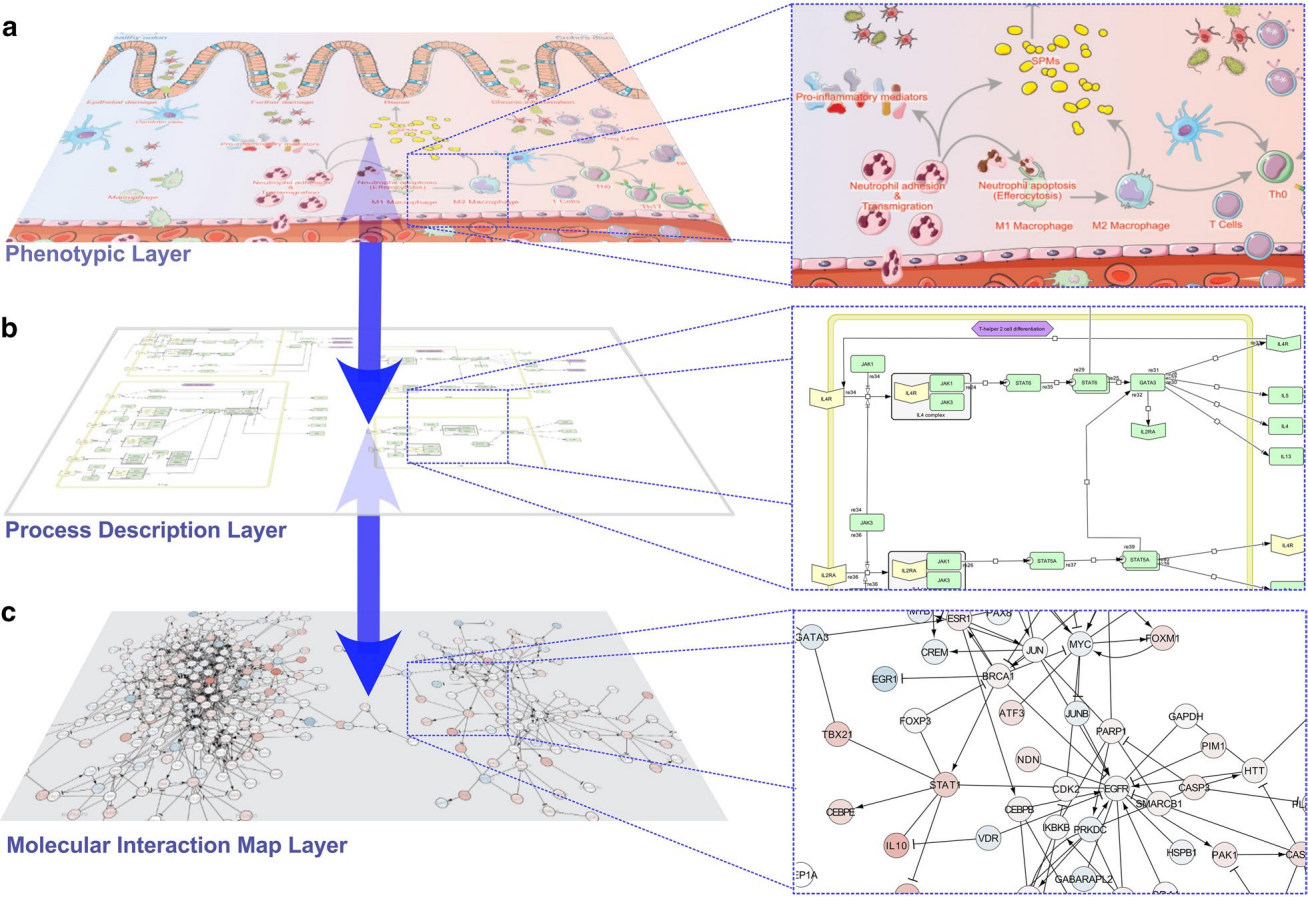
Hierarchal organization of an IBD disease map in three interconnected layers. **a** Representing the phenotypic layer to obtain an overview about the integrated content of the disease map. **b** Process description layer, **c** Molecular interaction map layer containing the relation of all involved genes for the complex disease investigation, in which the actual data can be integrated (colors can indicate different values, such as transcript expression, methylation ratio)

Since more heterogeneous high-throughput data are constantly generated, further integration of genomics, transcriptomics, epigenetics, as well as further omics data into a single framework is needed for an improved data interpretation. One possibility for such an overarching integration of complex data is the currently released disease map for acute inflammation resolution that may also serve as a prime example to facilitate the overall characterization of key-regulator processes in CD on a cellular level [53]. In particular, a complex disease phenotype, such as CD and IBD, can be comprehensively investigated considering the hierarchal organization of interacting components by a disease map (Fig. 1). In general, such a disease map can serve as an overarching knowledgebase to collect multiple kinds of data in a structured manner. In addition to a static data collection, such a framework offers the possibility for computational modeling to perform in silico experiments. For example, it would be possible to simulate knock-out gene experiments and retrieve the potential effect of the disease phenotype. This can be achieved because a disease map consists of three interconnected layers, which combine molecular pathway information, biological processes, and phenotypic outcomes. The top phenotypic layer highlights interactions at the tissue and cellular level, where a large number of immune cell types and tissue remodeling derive the clinical outcome (Fig. 1a). The middle layer describes connections between cellular processes and phenotypes (e.g., recognition of pathogens, invasion of neutrophils, resolution of inflammation, and efferocytosis). This layer also provides an interplay between key molecules associated with the underlying processes and resulting phenotypes (Fig. 1b). The computational simulations will be utilized within this layer by using all available information from the bottom layer. The bottom molecular interaction map layer contains all involved molecules and their regulatory components along with nonlinear regulatory motifs (e.g., feedback loops) and interactions (e.g., activation and inhibition of connected neighbor genes) that may control the overall dynamics of IBD (Fig. 1c). This information is usually gathered manually from domain experts to guarantee a well-curated basis for all analyses. The actual omics data are integrated and visualized in this layer, which means that specific colors in the nodes can indicate different values, such as transcript expression or methylation ratio. Taken together, such novel overarching computational concepts likewise facilitate the integration of diverse omics data and establish a profound knowledge base for CD.

## Epigenetic investigation in Crohn's disease

Several DNA methylation studies investigating CD-related epigenetic modifications are summarized in Tables 1 and 2.

### DNA methylation studies on blood components from Crohn's disease patients

Recent research has focused on the methylation landscape of the DNA from CD patients through epigenome-wide association studies (EWAS). DNA methylation studies on blood samples from CD patients with inactive or active disease are diverse and included analyses on whole blood samples [54], peripheral blood cells [55, 56], peripheral leukocytes [57–59], including peripheral blood mononuclear cells (PBMCs) [60, 61], serum samples [62], or separated B cells [63], T cells [64], and monocytes [65] (Table 1). The majority of studies involve adult patients [54–56, 58, 60, 62, 63, 65], while a few articles describe the DNA methylation in pediatric donors [57, 59, 61, 64]. A comparison between these studies is difficult because DNA methylation is strongly affected by age, or environmental conditions, such as medication or nutrition [66]. Interestingly, the advantage of studies using pediatric patients is that they are mostly less influenced by medication than adults suffering for a long time from IBD [67]. Thus, it is assumed that the epigenetic modifications more likely reflect the disease state. In addition to age, gender also influences methylation status, which is why some studies include only subjects of the same sex [54, 58, 65]. This leads to better comparability of results between patients and control subjects.

Cell-specific patterns in DNA methylation of CD patients were described in different subpopulations of blood cells [55, 63–65], enabling, for instance, the clustering of monocytes by their methylation profile [55]. Therefore, the validity of epigenetic analyses in whole blood samples is limited since individual cell-specific signals may disappear against the background of other present cells. For example, a study using CD patients found no different methylation profiles between CD patients and control subjects in whole blood samples. Still, in PBMCs from newly diagnosed pediatric CD patients the testis, prostate, and placenta-expressed protein *(TEPP)* gene showed CD-associated hypermethylation [59]. Interestingly, the effect was not detected in pediatric CD patients receiving therapy, indicating that the methylation status is associated with the disease activity [59]. This was confirmed by results in PBMCs, where the DNA methylation-related gene expression of homeobox protein engrailed-1 (*EN1*), Wilms tumor protein (*WT1*), and fibroblast growth factor receptor 2 (*FGFR2*) was higher in PBMCs from patients with active CD, while PR domain containing 16 (*PRDM16)* and neurogenic locus notch homolog protein 4 (*NOTCH4)* expression was decreased compared with patients in remission [68]. The altered expression of genes associated with the identified methylation-regulated gene region was further evaluated by gene expression analyses. Thereby, complement component 2 (*C2*), spondin-2 (*SPON2*), and lymphotoxin beta receptor (*LTBR*) were found to be increased in patients with active CD but not in patients in remission. Interestingly, in this study, the tumor necrosis factor alpha (*TNFA*) gene expression was increased in both groups, indicating a general altered CD-associated gene expression, which is not related to the disease activity. Although the results of PBMCs indicate that the disease activity in CD is associated with an altered methylation pattern, the epigenetic regulation of different subpopulations within PBMCs remains unclear.

Cell-specific studies identified hypermethylated and hypomethylated gene regions in monocytes of CD patients (*RPS6KA2* and *HCAC4*, respectively) but no corresponding modifications in CD4^+^ or CD8^+^ T cell populations [55]. The hypermethylation of the ribosomal protein S6 kinase alpha-2 (*RPS6KA2*) gene is assumed to result in a reduced gene expression of the encoded protein kinase. In tumor cell lines, the reduced or absent expression of *RPS6KA2* is associated with decreased apoptosis and increased proliferation [69], supporting earlier studies on the significance of *RPS6KA2* for cellular growth, survival, and differentiation [70]. In contrast, *HDAC4*, responsible for the deacetylation of lysine residues on the N-terminal part of the core histones (H2A, H2B, H3, and H4), is hypomethylated in monocytes from CD patients [55], resulting in an increased expression of the gene. The deacetylation of lysine by deacetylases is an epigenetic mechanism of histone modification and is generally assumed to inhibit gene transcription. However, potential targets of assumed deacetylase-induced gene repression in CD patients have still to be investigated. The fact that epigenetic modifications were described in monocytes of CD patients but not in T cells supports previous findings that transcriptional altered monocytes play a central role in CD pathogenesis that might be investigated in more detail by using single-cell technologies [71]. Interestingly, the methylation profile of CD14^+^ monocytes in female CD patients also differed at the level of the subpopulations (classical, intermediate, non-classical) [65], indicating different gene regulation according to the defined function of the respective subpopulation. However, on a genome-wide level, they did not identify statistically significant methylation differences when comparing CD patients vs. healthy subjects or CD patients with active disease vs. inactive disease patients [65], possibly due to the small number of subjects or high individual variances (Table 1).

Differences in the methylation status between blood-derived immune cells were further investigated in a comprehensive study using a large cohort of 240 newly diagnosed IBD patients [55]. Interestingly, in T cells from CD patients, tyrosine-protein kinase TXK *(TXK)* was identified as an immune-relevant target of cell-specific gene expression regulated by DNA methylation. Thereby, IBD-associated hypermethylation in the promoter region of *TXK* was negatively correlated with the gene expression in CD8^+^ T cells but not in other cell types indicating relations to underlying genotype. Due to the critical role of the *TXK* gene for regulation of the adaptive immune response, function, and differentiation of conventional T cells or non-conventional natural killer T (NKT) cells, the authors postulate a translational potential for the use of DNA methylation as biomarkers, capable of predicting the outcome or course of disease [55]. However, in newly diagnosed pediatric CD patients, the DNA methylation pattern in CD8^+^ T cells was only associated with age but not with the disease outcome [64]. A study on B cells from CD patients found 14 CD-specific CpG sites with altered methylation [63]. Several of the methylated loci found in CD are involved in immune system regulating pathways, like macrophage-stimulating protein receptor (*MST1R)*, interleukin-16 (*IL-16*), interleukin-10 (*IL-10*), and leukemia inhibitory factor (*LIF*) [63].

**Fig. 2:**
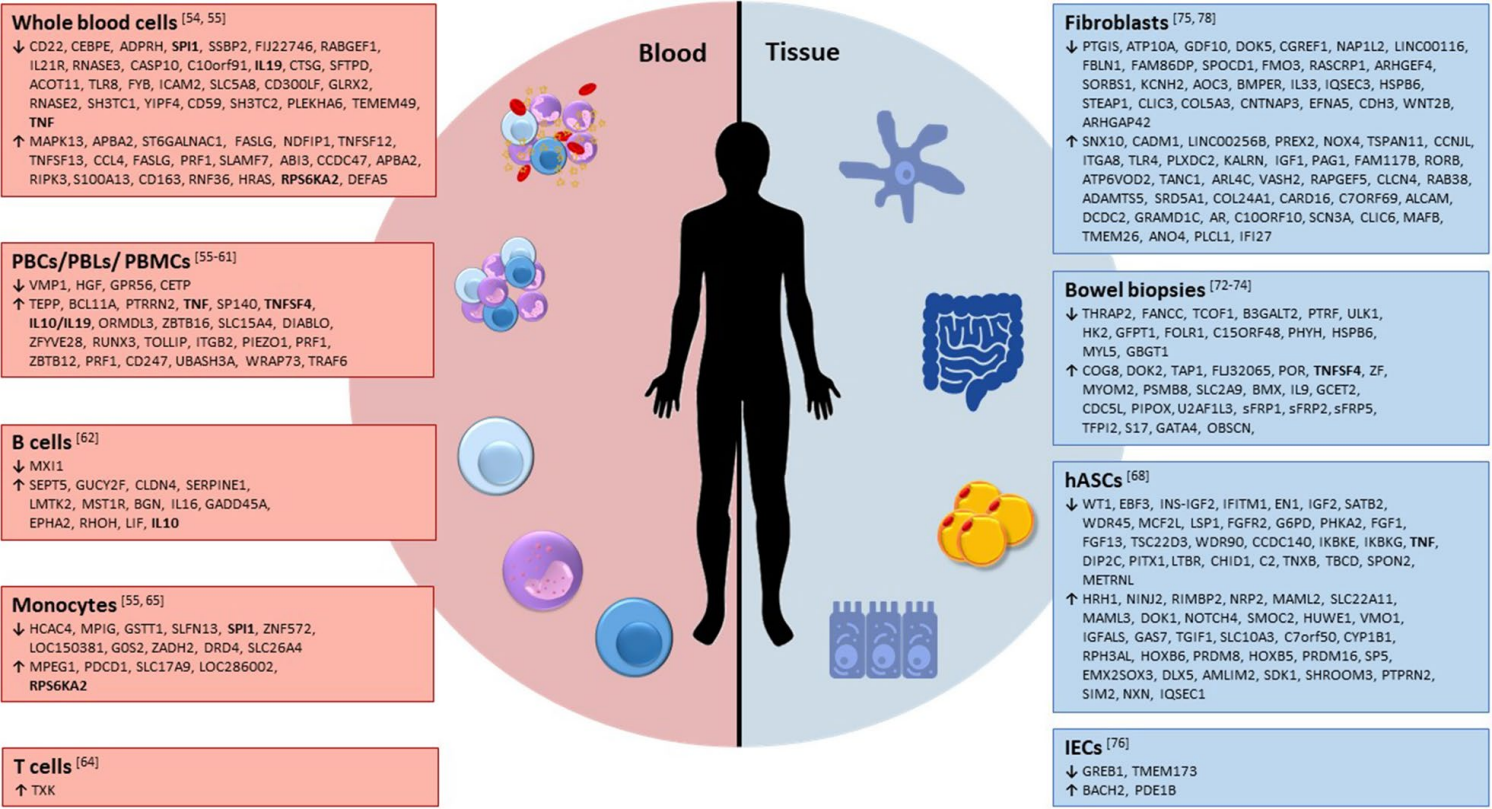
Schematic collection of DNA methylation studies in CD patients investigated in different cell types. Left/blood: hypo- (**↓**) and hypermethylated (**↑**) genes in whole blood cells, peripheral blood cells (PBCs), peripheral blood leukocytes (PBLs), peripheral blood mononuclear cells (PBMCs), B cells, monocytes, and T cells; Right/Tissue: hypo- (**↓**) and hypermethylated (**↑**) genes in bowel biopsies, isolated intestinal epithelial cells (IECs), and isolated fibroblasts from biopsies of CD patients and human adipose stem cells (hASCs) isolated from subcutaneous adipose tissue of CD patients. Genes found in more than 1 study are marked in bold

Moreover, methylated loci found in IBD, such as B cell lymphoma 3 protein (*BCL3*), signal transducer, and activator of transcription 3 (*STAT3*), oncostatin-M (*OSM*), and signal transducer and activator of transcription 5 (*STAT5*), are involved in regulation or downstream signaling in the Interleukin-23 (IL-23) signaling pathway [72]. IL-23 is a heterodimeric cytokine with functions in innate and adaptive immunity that induces autoimmune inflammation and thus may be responsible for the pathogenesis of autoimmune inflammatory diseases, such as CD. Besides, several studies on blood samples from CD patients show DNA methylation alterations mostly in genes involved in immune-related pathways like immune system progresses, immune response, or defense response to bacteria [54–56, 58, 63, 65] (Fig. 2). However, due to the limited sample size and the minor effect sizes observed, many of the results have to be validated in larger cohorts to evaluate their potential as valuable biomarkers.

### DNA methylation studies on tissue components from Crohn's disease patients

Whole intestinal biopsies [67, 73, 74] or cells, isolated and cultured from intestinal biopsies [68, 75–79], were used to investigate potential CD-associated epigenetic modifications in gut tissue samples derived from pediatric [67, 76, 79] or adult patients [68, 73–75, 77, 78]. An overview of the identified methylation-regulated genes, described in association with CD, is presented in Fig. 2. A study on intestinal epithelial cells (IECs) of human fetuses, healthy pediatric donors, and children newly diagnosed with CD displayed general differences between fetal and pediatric samples, indicating that DNA methylation is involved in the maturation and development of gut epithelium [79]. Furthermore, Kraiczy et al. postulate that epigenetic changes during critical periods of fetal and postnatal development may predispose the onset of chronic intestinal inflammation. In children newly diagnosed with CD, hypermethylated regions were identified in immune relevant genes, such as mucin (*MUC2*) and polymeric immunoglobulin receptor (*PIGR*), indicating a suppressed gene transcription. Interestingly, clustering analysis of methylation profiles between IBD (including both CD and ulcerative colitis (UC) patients) and healthy controls revealed that the subset of IBD appeared not to be entirely due to the presence of inflammation, which was assumed to reflect an IBD-specific intestinal epithelial DNA methylation signature in these patients [79].

When comparing inflamed and non-inflamed biopsies of adult CD patients, it is not surprising that specific IBD associated genes are altered in their methylation state, for example, unc-51 like autophagy activating kinase 1 (*ULK1)*, docking protein 2 (*DOK2)*, and antigen peptide transporter 1 (*TAP1)* [74]. *ULK1* is an essential protein in autophagy in response to starvation [72, 80] and hypermethylated in inflamed rectal biopsies from CD patients compared with controls, which means that the *ULK1* gene expression is decreased [74]. It is well described that autophagic dysfunctions play multiple roles in the pathogenesis of IBD and, in particular, in CD [81, 82]. Also, several genetic variants are linked to CD, such as nucleotide-binding oligomerization domain-containing protein 2 (*NOD2)* or autophagy-related protein 16–1 (*ATG16L1)* [81]. In contrast to *ULK1, DOK2* and *TAP1* are hypomethylated in CD patients, which leads to an increased gene expression. Both genes are involved in inflammation-related immune response, such as cytokine- or antigen-induced signaling, respectively [72]. The association between inflammation and methylation state was further confirmed in studies investigating epigenetic modification by medication. Thereby, differences in DNA methylation between previously inflamed tissue of CD patients and healthy subjects were gone after medicinal treatment [74], indicating a direct effect of acute inflammatory mediators on epigenetic modulation.

Researchers investigated 236 IEC samples from differently localized mucosal biopsies of the intestine from pediatric patients with IBD or healthy controls to evaluate the role of methylation independently of the inflammatory status [76]. In this context, segment-specific DNA methylation differences were described in the region of terminal ileum between CD patients and healthy controls, indicating CD-associated epigenetic patterns. Interestingly, these changes appear to be specific to CD, as no differences were found in UC. In contrast, the authors described the epigenetic changes observed in the colon as common IBD signature because the differences in DNA methylation occurred in CD patients and in patients with UC. This suggestion is supported by the investigation of the DNA methylation landscape in colon biopsies from pediatric UC and CD patients. They showed similar DNA methylation alterations in 50% of the verified genes between CD and UC patients [67].

A serious complication of CD is fibrosis-associated strictures, which may cause intestinal obstruction. Epigenetic studies have shown that alterations in the DNA methylation landscape are associated with fibrosis-associated complications in CD patients [75, 78]. When comparing biopsy samples of non-inflamed (NINF) tissues, inflamed tissues (INF), and stenotic tissues (STEN) from CD patient biopsies with control subject samples, in one study, 44 different methylated positions (DMPs), 38 different methylated regions (DMRs), and 11 different expressed genes (DEGs) were found [78]. However, most methylation differences occurred when comparing CD-INF with CD-NINF or CD-STEN with CD-NINF [78]. A further study detected 1,180 DMRs as hypermethylated and 802 DMRs as hypomethylated in the fibrotic samples compared to unaffected samples [75]. Furthermore, the authors found that the majority of differential DNA methylation was within introns (48.6% hypermethylated, 43.1% hypomethylated) and intergenic regions (40.8% hypermethylated, 48.4% hypomethylated), which are involved in the regulation of gene transcription in addition to gene-associated promoters. For instance, DNA methylation in introns can modulate alternative exon splicing, while intergenic sequences contain enhancers and insulators associated with the regulation of gene expression [83, 84]. In gene promoter regions, only 5% of the DMRs were hypermethylated and 2.7% hypomethylated [75]. Both studies on fibrosis-associated complications observed downregulation of the *WNT* signaling pathway member *WNT2B*, which was correlated with increased DNA methylation [75, 78]. During intestinal inflammation, *WNT2B* and other canonical *WNT* ligands, such as *WNT1*, *WNT3A*, and *WNT10A*, are produced by intestinal macrophages [85–87], mucosal dendritic cells [88], and T cells [89] and secreted to activate *WNT*/β-catenin signaling in crypt base columnar (CBC) stem cells, thereby promoting epithelial regeneration after tissue damage [90]. Accordingly, it can be assumed that an interruption of the signal harms mucosal healing and may contribute to chronic inflammation [90]. In addition to *WNT*-signaling, multiple components of the E2 factor family of transcription factors (*E2F)* and fibroblast growth factor (*FGF)* pathway were observed to be downregulated by increased DNA methylation in biopsies samples from CD patients. Since *E2F*, *WNT*, and *FGF* signaling pathways are essential for cellular differentiation, proliferation, and migration, it can be hypothesized that their abnormal behavior underlies tissue remodeling processes, resulting in the observed fibrostenotic phenotype [78].

In addition to intestine-derived samples, one recent study examined the DNA methylation status of human adipose stem cells (hASCs) from CD patients [68] as they showed dysfunction in proliferation, lipid enrichment, and immunomodulation in quiescent CD [77]. Several different methylated and expressed genes (Fig. 2) were involved in immune system responses, metabolism, cell differentiation, and development processes [68]. Interestingly, they also compared the methylation and gene expression results from hASCs with PBMCs from patients with active and inactive CD and found that genes, which are differentially methylated in both cell subsets, were related to the immune system and cell differentiation processes. Furthermore, the study showed that changes in DNA methylation-related gene expression in hASCs are only partially restored in quiescent CD, which was similar to the effects observed in PBMCs [68]. This suggests that epigenetic modifications may induce the persistence of latent inflammation against a background of apparent clinical remission.

### Histone modification studies in Crohn's disease patients

In contrast to global methylation patterns, histone modifications have been less extensively studied in CD than DNA methylation. A recent study found that the histone methylation profile at H3K4 changes when the microbiome altered resulting from the disease [91]. The epigenetic changes are related to genes involved in regulating cytokine signaling, metabolism, homeostasis, and reactive oxygen species [91]. A significant proportion of the analyzed genes had trimethylation of H3K4 (H3K4me3), one of the least abundant histone modifications and positively correlates with transcription [92]. In CD patients, levels of H3K4me3 correlated with the severity of bowel inflammation, although significant differential expression was lacking [91]. Moreover, Turgeon et al. showed that H3K27 marks might control both cell proliferation and inflammatory response by regulating specific signaling pathways, either negatively for the STAT3 pathway or positively for the p38 mitogen-activated protein kinase (MAPK) pathway [93]. Even the slightest modifications in polycomb repressive complex 2 (PRC2) activity lead to variations in H3K27 methylation and to activation or repression of parallel pathways of gene activity. In the context of genetic disorders, this results in both increased proliferation and intrinsic inflammatory gene stimulation. Furthermore, in two animal models for intestinal inflammation and intestinal biopsies of patients with CD, it was shown that the acetylation of histone H4 in the inflamed mucosa was significantly increased in the trinitrobenzene sulfonic acid model of colitis, especially on lysine residues 8 and 12 in contrast to non-inflamed tissue [94], indicating an activation of the related gene region. Besides, acetylated H4 was localized on inflamed tissue and Peyer's patch during dextran sulfate sodium (DSS)-induced colitis in rats, and H4 acetylation was also significantly upregulated in inflamed biopsies and on the Peyer's patch in patients with CD. Another study found 56 potential target genes for H3K27ac change in colon tissue from mice upon DSS treatment [95]. It can therefore be assumed that histone acetylation is associated with intestinal inflammation in CD patients and may represent a new therapeutic target for mucosal healing [94].

## Epigenetic signatures as biomarkers in CD

Changes in the DNA methylation status of CD-associated genes significantly alter transcriptional activity and expression levels of genes, thereby influencing disease risk and progression. Interestingly, some DNA methylation profiles apply to both CD and UC, while others are specific to CD or UC, creating new and meaningful rationales for disease classification and therapy [74]. Also, epigenetic analysis has found several differentially methylated genes involved in IBD that were not previously identified as IBD risk genes in GWAS.

The diagnosis of IBD is primarily based on clinical imaging, as well as endoscopic and histological findings. Furthermore, the emergence of genetic, serological, and fecal markers in the diagnosis and classification of the disease is also growing. Especially in the differentiation between UC and CD, biomarkers can be of high relevance for an early, correct diagnosis and optimal therapy. Despite all these possibilities, some patients are still diagnosed with “IBD-unclassified” or “indeterminate colitis” because a clear assignment to CD or UC is not possible. Molecular markers, such as DNA methylation and histone modifications, could have certain advantages in terms of sensitivity, specificity, and accuracy when used in conjunction with other surrogates, such as *NOD2*, anti-*Saccharomyces cerevisiae* antibodies (ASCA), anti-neutrophil cytoplasmic antibodies (ANCA), fecal calprotectin (FC), and fecal lactoferrin (FL) [96, 97]. At the epigenetic level, microRNAs in particular currently assume a central role in biomarker development in CD, compared with DNA methylation and histone modifications [98].

As shown in Fig. 2, a subset of genes are found to be differentially methylated in independent studies, for example, *TNF*, Tumor Necrosis Factor Ligand Superfamily Member 4 (*TNFSF4*), *IL-19*, *IL-10*, *RPS6KA2*, and Spi-1 proto-oncogene (SPI1) (Fig. 2). Hypomethylation of *TNF* and consequent increased gene expression, and hypermethylation of *TNFSF4* and consequent repressed gene expression, were found in both blood and tissue samples, whereas hypermethylation of *IL-10* and *RPS6KA2*, as well as hypomethylation of *SPI1*, could only be detected in blood samples from CD patients. *TNF*, as a pro-inflammatory cytokine, plays an essential role in local and systemic inflammation, so that activation of gene expression in CD is not surprising. Increased *TNF* secretion was found deeper in the *lamina propria* and submucosa of CD patients [99–101]. Furthermore, *TNF* levels were elevated in stool samples from patients with active CD compared with inactive CD patients and healthy controls, although serum concentrations were less pronounced [101–104]. However, due to the multifunctional mode of action of *TNF*, methylation change is not suitable as a biomarker for the identification of CD but only shows whether inflammation is present or not.

Different DNA methylation of *SPI1* and *RPS6KA2* has been demonstrated in both whole blood and isolated monocytes. *SPI1* is responsible for developing myeloid and B-lymphoid cells [105], whereas *RPS6KA2* regulates different cellular processes, including cell growth, cell motility, proliferation, and cell cycle progression [55, 106]. Presumably, the altered DNA methylations are cell-specific aspects that tend to be involved in the development and differentiation of monocytes themselves rather than CD's pathogenesis. Future studies would have to take into account that different cell types may have diverse DNA methylation landscapes, which is crucial to decipher and elucidate the exact functional consequences of epigenetic changes in IBD pathogenesis. Another limitation points to results obtained in studies with small sample sizes that have to be verified in large, well-designed, prospective studies.

## Conclusions

Our knowledge of the functional significance of epigenetic modifications in CD is still limited, although EWAS have contributed to a better understanding of the pathogenesis of IBD. Most of the described studies examined DNA methylation changes between patients and control subjects to find biomarkers for an early IBD identification, or more specifically, to enable a differential diagnosis between CD and UC. However, specific patterns of DNA methylation and histone modifications might be used as alternative biomarkers of disease activity or progression and novel targets for therapeutic interventions in IBD patients. Thus, it will be necessary to decipher and elucidate the precise functional consequences of genetic and epigenetic alterations in CD's pathogenesis to pave the way for the development of novel therapeutic strategies. The fact that different cell types have diverse DNA methylation landscapes has to be considered. Moreover, it should be taken into account that the epigenetic changes may be reversible, for example, by drugs, and are caused not only by the disease status but also by environmental factors, which, however, influence the pathogenesis of the disease. The questions at that point are: [1] Is the epigenetic modification altered due to the disease or is the disease driven due to the epigenetic modification, and (2) how do epigenetic modifications contribute to the pathogenesis of CD? Are there CD-specific signatures, which provide additional value as disease-related biomarkers or serve as targets for therapeutic interventions? Epigenetic methods opened up a broad field of research to answer these questions and improve our knowledge of the immune dysregulation that leads to CD. Computational models based on experimentally identified biomarkers may help clinicians for an improved disease characterization and/or predict the disease's course. Since epigenetic modifications are dynamic and reversible, they could ultimately serve as novel essential therapeutic targets.

## Data Availability

Not applicable.

## Abbreviations

ANCA: Anti-neutrophil cytoplasmic antibodies
ASCA: Anti-*Saccharomyces cerevisiae* antibodies
ATAC-Seq: Assay for transposase accessible chromatin with high-throughput sequencing
ATG16L1: Autophagy-related protein 16-1
BCL3: B cell lymphoma 3 protein
bp: Base pair
C2: Complement component 2
CBC: Crypt base columnar
CD: Crohn's disease
CD14^+^: Cluster of differentiation 14
ChIP-Seq: Chromatin immunoprecipitation followed by DNA-sequencing
CpG: Regions of DNA where a cytosine nucleotide is followed by a guanine nucleotide
DEFA5: Defensin alpha 5
DEG: Different expressed gene
DL: Deep learning
DMP: Different methylated positions
DMR: Different methylated regions
DNMT: DNA methyltransferases
DOK2: Docking protein 2
E2F: E2 factor family of transcription factors (e.g., E2F1, E2F2, E2F3a)
EN1: Homeobox protein engrailed-1
ENCODE: Encyclopedia of DNA elements
eQTL: Expression quantitative trait loci
EWAS: Epigenome-wide association study
FC: Fecal calprotectin
FGF: Fibroblast growth factor
FGFR2: Fibroblast growth factor receptor 2
FL: Fecal lactoferrin
GWAS: Genome-wide association study
hASC: Humane adipose stem cells
IBD: Inflammatory bowel disease
IEC: Intestinal epithelial cell
IL-10: Interleukin-10
IL-16: Interleukin-16
IL-23: Interleukin-23
INF: Inflamed
LIF: Leukemia inhibitory factor
LTBR: Lymphotoxin beta receptor
MAPK: Mitogen-activated protein kinase
miRNA: MicroRNA
ML: Machine learning
MSP: Methylation-specific PCR
MST1R: Macrophage-stimulating protein receptor
NINF: Non-inflamed
NKT: Natural killer T cells
NOD2: Nucleotide-binding oligomerization domain-containing protein 2
NOTCH4: Neurogenic locus notch homolog protein 4
OSM: Oncostatin-M
PBC(s): Peripheral blood cell(s)
PBMC(s): Peripheral blood mononuclear cell(s)
PCR: Polymerase chain reaction
PRDM16: PR domain containing 16
RPS6KA2: Ribosomal protein S6 kinase alpha-2
Seq: Sequencing
SPON2: Spondin-2
STAT: Signal transducer and activator of transcription
STEN: Stenotic
TAP1: Antigen peptide transporter 1, ATP binding cassette subfamily B member
TCERG1L: Transcription elongation regulator 1-like protein
TEPP: Testis, prostate and placenta-expressed protein
TET: Ten-eleven translocation enzymes
TNF: Tumor necrosis factor
TSG: Tumor suppressor genes
TXK: Tyrosine-protein kinase TXK
UC: Ulcerative colitis
ULK1: Unc-51 like autophagy activating kinase 1
WNT: WNT family member protein, transmembrane receptors
WT1: Wilms tumor protein
